# Cross-cultural adaptation and content validity of leisure attitude measurement for older adults

**DOI:** 10.11606/s1518-8787.2020054002373

**Published:** 2020-11-12

**Authors:** Vivian Carla de Castro, Fernanda Shizue Nishida, Flavia Maria Derhun, Anai Adario Hungaro, Eraldo Schunk Silva, Lígia Carreira

**Affiliations:** I Centro Universitário Integrado Faculdade de Medicina Campo MourãoPR Brasil Centro Universitário Integrado. Faculdade de Medicina. Campo Mourão, PR, Brasil; II Centro Universitário de Maringá Departamento de Medicina MaringáPR Brasil Centro Universitário de Maringá. Departamento de Medicina. Maringá, PR, Brasil; III Universidade Estadual de Maringá MaringáPR Brasil Universidade Estadual de Maringá. Programa de Pós-Graduação em Enfermagem. Maringá, PR, Brasil; IV Universidade Estadual de Maringá Departamento de Estatística MaringáPR Brasil Universidade Estadual de Maringá. Departamento de Estatística. Maringá, PR, Brasil; V Universidade Estadual de Maringá Departamento de Enfermagem MaringáPR Brasil Universidade Estadual de Maringá. Departamento de Enfermagem. Maringá, PR, Brasil

**Keywords:** Aged, Leisure Activities, Attitude to Health, Validation Study

## Abstract

**OBJECTIVE:**

To adapt the Leisure Attitude Measurement to the Brazilian culture and to evaluate the face and content validity of the Brazilian version for older population.

**METHODS:**

Methodological study of cross-cultural adaptation in five stages: initial translation; synthesis of translations; back translation; evaluation by a committee of experts using a face and content validity assessment instrument; pre-test with 36 elderly, selected by convenience, with the application of a pre-test evaluation instrument. Data were analyzed descriptively and internal consistency measured by Cronbach's alpha coefficient.

**RESULTS:**

Evidenced face and content validity of the adapted version, as well as its equivalence with the original version. In the pretest, the elderly were 71.5 years old on average, 66.7% were women, 47.2% had a stable union, 66.7% lived with family members, 47.2% had 12 or more years of education and 58.3% received two minimum wages or more. The instrument revealed good internal consistency with a coefficient of 0.95 for the total global instrument and 0.88, 0.92 and 0.88 for the cognitive, affective and behavioral domains, respectively.

**CONCLUSIONS:**

The instrument's adaptation to the Brazilian culture was successful and allows to assess the attitude of the elderly in relation to leisure in a reliable manner, even though the results are a preliminary version, to be concluded after the psychometric analysis. The instrument could be incorporated in various health fields in Brazil and will allow the production of standardized data, comparison between cultures and strategies to promote positive attitudes towards leisure.

## INTRODUCTION

Older adults constitute a rapidly and substantially growing segment of the population worldwide, with estimates that draw attention by pointing to a total of 1.2 billion people aged 60 and over in 2025 ^1^ . If, on the one hand, these data show an opportunity for advancement in terms of quality and life expectancy, on the other hand, they create uncertainties regarding the social, financial and health maintenance of this age group ^2^ . Because this phase is usually associated with the exit of the individual from the labor market, it is marked by physical, psychological, social and mental changes, which may lead to the loss of personal identity and interfere with quality of life ^3^^,^^4^ .

It is critical for the successful aging to keep an active mind and body ^5^ , whether by investing in engaging in occupational and social activities or autonomy and well-being ^2^ . In this sense, the literature deals with the benefits that leisure represents as a strategy to cope with the challenges, losses and disabilities in old age ^6^^,^^7^ . Here, leisure is understood as a set of experiences that can develop in many cultural contents and which expresses itself in the time available beyond professional obligations, in a free and authentic way, and the individual can actually enjoy idleness ^8^ .

In the health field, involvement in leisure activities promotes physical health, positive social interactions, psychological well-being and quality of life ^5^^,^^7^^,^^9^ . Thus, leisure can be considered as a conditioner and determinant of the health-disease process, especially regarding mental health ^10^ . In addition, involvement in leisure activities is a predictor of health self-perception and functional capacity in the older adults, and some leisure activities are related to health-promoting behavior ^11^ .

People's practices during leisure says more about their attitude than in any other context ^4^^,^^10^^,^^12^ . Therefore, it is relevant to know the attitude of the older people in relation to leisure in order to allow a better understanding of this type of social practice and stimulate more positive attitudes and engagement in leisure activities ^6^^,^^13^ .

Based on the concepts of social psychology, attitude is a system of knowledge and beliefs, provided with feelings, for or against a particular social object, that generates the intention of attitude on the knowledge and feelings related to that object. Thereby, it is a multidimensional construct that aggregates cognitive, affective and behavioral components, developed from individual characteristics, learning processes, environmental influences and previous experiences ^14^ . This way, the person's attitude can change over time, while acquiring new experiences and assimilating new knowledge, as happens during the aging process ^13^ .

To assess the attitude of the older people in relation to leisure, it is essential to use reliable instruments whose results allow the elaboration of health strategies to be appropriate to reality. Leisure Attitude Measurement (LAM) is one of these instruments and aims to measure cognitive, affective and behavioral leisure-related attitude ^15^ . It has been adapted and validated for other countries such as China ^16^^,^^17^ , Malásia ^18^ , Portugal ^19^^,^^20^ and Turkey ^21^ .

Leisure is not experienced in the same way by all individuals; after all, there is no single appropriate approach to explain the social structures and values related leisure in other countries and cultures ^12^ . Given this, cross-cultural adaptation emerges as an alternative to the development of a new instrument because it requires less resources and favors the comparison between different contexts and populations ^22^ .

Considering that cross-cultural adaptation procedures have increasing emphasis as an instrument for the development of public health practice and science ^22^^,^^23^ , the aim of this study was to adapt the Leisure Attitude Measurement to the Brazilian culture and assess the face and content validity of the Brazilian version for older adults.

## METHODS

This is a methodological study of cross-cultural adaptation. Authorization was obtained from one of the authors of the instrument, as well as from the copyright holder, through e-mail, for cross-cultural adaptation. The translation and adaptation process of the instrument was outlined in five steps ( Figure ): 1) Initial translation into the target language; 2) Synthesis of translations; 3) Back-translation to the original language; 4) Evaluation by a committee of experts; 5) Pre-test ^24^ .

**Figure:**
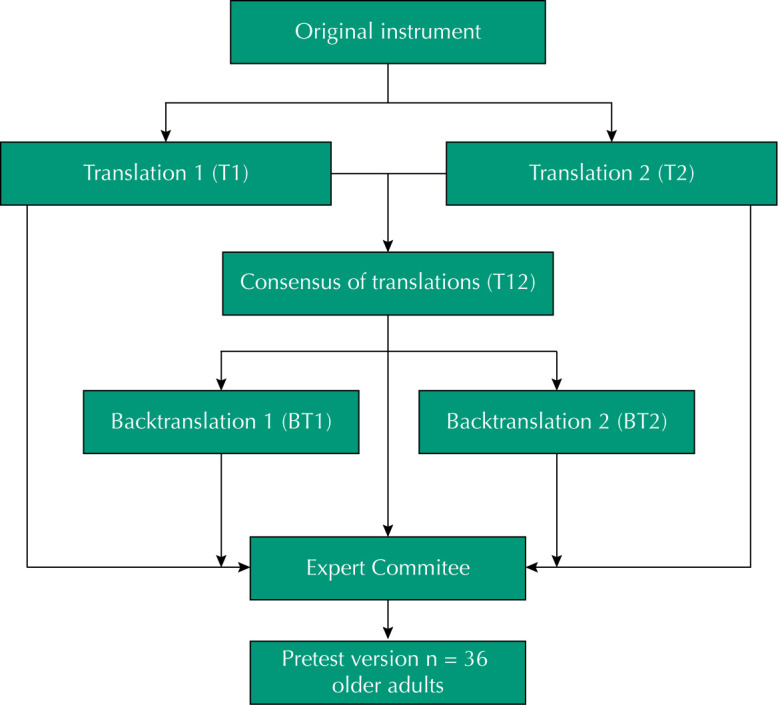
Flowchart of steps of cross-cultural adaptation of Leisure Attitude Measurement.

The original version of LAM consists of 36 items, divided into three domains related to attitude topics, with 12 items each. A five-level Likert scale is used as a response system (“1” = extremely negative attitude, “5” = extremely positive attitude, “3” = midpoint) and scores are obtained by arithmetically adding the responses to the items. For the separate domains, the midpoint is 36 and for the global instrument is 108. Values above this point reveal positive attitudes and, below, negative attitudes towards leisure ^20^ .

Two translations (T1 and T2) of the LAM into Portuguese were performed by two independent translators, both Brazilians and mastery of American culture and English language, and only one of them was informed about the objectives of the instrument and the study. The two translated versions (T1 and T2) were created into a synthesis version (T12), based on consensus between the translators, through a single virtual meeting lasting approximately forty minutes. On that occasion, the discussion about the differences, changes and comments regarding the translations was mediated by the researcher. In the third stage, T12 was back-translated (RT1 and RT2) by two American translators, with mastery of the Portuguese language and Brazilian culture, in which both were unaware of the objectives of the study. The Brazilian and American translators were selected by a company specializing in certified translations. The three stages took place between August 2017 and February 2018.

For the committee of experts, were invited four bilingual (Portuguese/English) health professionals and an English teacher, who was trained and specialized in the health area, working as an English teacher in regular and language schools. Of the four healthcare professionals, one is a PhD in Physical Education, a professor with extensive experience in assessing the psychometric properties of measuring instruments; three are nurses, two PhDs and one PhD student in Nursing, all professors, one with experience in evaluating the psychometric properties of measuring instruments and the health of the older population, and the other with experience in quantitative research methodologies and experiences in older adults health. After accepting to participate in the study, the experts received all the material by email, which included the two translations, the synthesis version and the two back-translations, as well as an instrument developed by the researcher to perform face and content validation of LAM. The committee review process took place between March and May 2018.

To assess semantic, idiomatic, conceptual and cultural equivalence between the original and the adapted instrument, the specialists received guidelines to fill out the instrument for evaluating the face and content validity. According to the adopted protocol ^19^ , in semantic equivalence, the meaning of words was assessed through vocabulary and grammar; in idiomatic equivalence, the colloquial expressions used were analyzed; in conceptual equivalence, it was about the importance of the items and aspects that were addressed; and, in cultural equivalence, it was observed whether the items captured and measured the daily life experiences of the target audience. Each specialist analyzed one item at a time, compared to its original version, and indicated whether it was equivalent, partially equivalent or not equivalent between the two versions, using different fields for semantic, idiomatic, conceptual and cultural aspects. In cases of partial equivalence or non-equivalence between items, experts were instructed to suggest adjustments.

The Content Validity Index (CVI) of each item was also assessed by applying a four-point likert scale, in which experts scored “1” if not relevant, “2” if item needed major revision to be relevant, “3” if item needed minor revision to be relevant or “4” if relevant item. The score was calculated by the sum of agreement of the items marked by “three” or “four” by the experts, divided by the total number of answers, being considered appropriate those items with agreement rate higher than 80% ^25^ .

The pre-test version of Brazilian LAM was based on the expert's suggestions and, one more time, sent to them for final opinion. Once approved, a pre-test was carried out with 36 seniors who attended the University of the Third Age (UnATI), a project from the State University of Maringá. Data collection took place in two weekdays, when there were classes, in June 2018, and the sample was selected by convenience. After reading and answering to the pre-test version of the Brazilian LAM, the older people filled out a pre-test evaluation instrument, developed by the researcher, in order to assess the clarity and understanding of the items. This instrument had six questions, a space for suggestions and variables for participant's sociodemographic characterization, namely: age, gender, educational level, income and family arrangement.

Data were described using simple frequency tables. The internal consistency of the instrument for the 36 items analyzed was measured using Cronbach's alpha coefficient. Cronbach's alpha with values above 0.7 was considered ideal. Data were analyzed using the Statistical Analysis Software Program (SAS, version 9.4) from a spreadsheet created in the Excel program.

Ethical approval for the study was granted by the Human Research Ethics Committee of the State University of Maringa (COPEP/UEM) (protocol 2,194,308/2017).

## RESULTS

Of the 36 items translated in the first stage, 18 (50%) showed some discrepancy in T1 and T2, the main ones being related to similar words or phrases in Brazilian Portuguese (eg “quite” and “considerable”; “some” and “way”) and use of first or third person pronouns (“make us” and “make the person”). The other half of the items were identical in the translations, with six belonging to the cognitive domain, seven to the affective domain and five to the behavioral domain.

Inthe synthesis of the translations, discrepancies with the translators were resolved, opting for the expressions or words that provided the item with greater clarity. It was also considered, regarding the older people community in the Brazilian context, to discuss the exchange of some words from items that were identical in T1 and T2 for more popular terms in Brazilian Portuguese, without modifying the meaning expressed in the item, for better the understanding of respondents, namely: “self-development” exchanged to “personal development”; “waste” exchanged to “spend”; “had time available” exchanged “had time to spend”. In the back-translated versions, 27 items (75%) were identical, and the words that differed between RT1 and RT2 in the remaining nine items were considered synonymous, showing that the T12 version corresponded to the original version.

In the fourth stage, the experts evaluated the equivalence of the items, the statement and the measurement scale of the Brazilian version of LAM (LAM-BV), as well as the relevance of each item, calculated using the CVI. The CVI was excellent (1.0) for 31 items and acceptable (0.8) for five items, requiring adjustments. The agreementpercentage of the experts was 86.2%.

A total of 69 suggestions were made by the five experts, which considered 16 (44.5%) items equivalent to the original version in all aspects. Another 20 (55.5%) items were partially equivalent, and of these, eight received this rating from at least two experts and two items were concomitantly assessed as non-equivalent, in at least one aspect by one of the experts. Table 1 pointed out these 20 items and their respective CVI, totaling 23 partial equivalences in semantic, 11 in idiomatic, nine in conceptual and ten in cultural, and two non-equivalences in idiomatic and one in semantic, conceptual and cultural aspects.

**Table 1:** Partial equivalences and non-equivalences between items of the original and adapted versions of LAM, in the semantic, idiomatic, conceptual and cultural aspects, according to the experts and their respective CVI. Maringá, Paraná, Brazil, 2018.

Itens		Expert 1	Expert 2	Expert 3	Expert 4	Expert 5	IVC
Cognitive domain							
	3		P a	P a , b , c , d		P d	1.0
	4	P b , c					1.0
	5	P a , b	P a				1.0
	8		P a			P a	1.0
	11		P a				1.0
Affective domain							
	13	P a , b , c , d	P a				0.8
	16					P d	1.0
	17		P a				1.0
	19	N a , b , c , d				P a	0.8
	21	P a , b , c , d					0.8
	22	P a , b , c , d					1.0
	24	P a , b , c , d				P b	1.0
Behavioral domain							
	26		P a				1.0
	28		P a				1.0
	29		P a				1.0
	30	P b	P a				1.0
	32		P a				1.0
	33		P a				1.0
	35	N b /P a , c , d				P d	0.8
	36	P b , c	P a			P a , b , c , d	0.8

LAM = Leisure Attitude Measurement. CVI =Content Validity Index. P = partial equivalence of the item in the original and adapted versions. N = non-equivalence of the item in the original and adapted versions. CVI values above 0.8 are considered adequate.

a Semantics.

b Idiomatic.

c Conceptual.

d Cultural.

The main suggestions for the partially equivalent or non-equivalent items were to remove the word “time” from the expression “leisure time” in items 3 and 16; add the word “individual” or “person” in items 4, 6 and 7; exchange “development” by the word “improvement” in item 8; use “get involved” instead of “participate” in items 13, 19 and 34, considering the legitimate translation of the original version and the deeper meaning of “get involved” in the Brazilian context; to replace the verb“spend”by using“I dedicate” in items 22 and 35, since “spending” may have a negative meaning; to exchange the expression “I do not like to be in a hurry” to “I like to enjoy every moment” in item 19; replace “environment” with “place” in item 30, as it is the most used expression in the Brazilian context. Box shows the original and final versions after the cross-cultural adaptation process.

**Box:** Translation and cross-cultural adaptation of Leisure Attitude Measurement to Brazilian Portuguese.

Item	Original version – Leisure Attitude Measurement	Final version – Leisure Attitude Measurement - Brazilian Version
1	Engaging in leisure activities is a wise use of time.	*Participar de atividades de lazer é uma forma inteligente de utilizar o tempo* .
2	Leisure activities are beneficial to individuals and society.	*As atividades de lazer são benéficas para os indivíduos e para a sociedade* .
3	People often develop friendships in their leisure.	*As pessoas costumam fazer amizades durante o lazer* .
4	Leisure activities contribute to one's health.	*As atividades de lazer contribuem para a saúde dos indivíduos* .
5	Leisure activities increase one's happiness.	*As atividades de lazer deixam as pessoas mais felizes* .
6	Leisure increases one's work productivity.	*O lazer aumenta a produtividade dos indivíduos no trabalho* .
7	Leisure activities help to renew one's energy.	*As atividades de lazer ajudam a renovar as energias das pessoas* .
8	Leisure activities can be a means for self-improvement.	*As atividades de lazer podem ser uma forma de aperfeiçoamento pessoal* .
9	Leisure activities help individuals to relax.	*As atividades de lazer ajudam os indivíduos a relaxar* .
10	People need leisure activities.	*As pessoas precisam de atividades de lazer* .
11	Leisure activities are good opportunities for social contacts.	*As atividades de lazer são boas oportunidades para o convívio social* .
12	Leisure activities are important.	*As atividades de lazer são importantes* .
13	When I am engaged in leisure activities, the time flies.	*Quando estou envolvido(a) em atividades de lazer, o tempo passa rápido* .
14	My leisure activities give me pleasure.	*Minhas atividades de lazer me dão prazer* .
15	I value my leisure activities.	*Valorizo as minhas atividades de lazer* .
16	I can be myself during my leisure.	*Sinto-me à vontade durante meu lazer* .
17	My leisure activities provide me with delightful experiences.	*Minhas atividades de lazer me proporcionam experiências prazerosas* .
18	I feel that leisure is good for me.	*Sinto que o lazer é bom para mim* .
19	I like to take my time while I am engaged in leisure activities.	*Gosto de aproveitar cada momento quando estou envolvido(a) em atividades de lazer* .
20	My leisure activities are refreshing.	*Minhas atividades de lazer são revigorantes* .
21	I consider it appropriate to engage in leisure activities frequently.	*Considero apropriado participar de atividades de lazer com frequência* .
22	I feel that the time I spend on leisure activities is not wasted.	*Sinto que o tempo que utilizo em atividades de lazer não é tempo perdido* .
23	I like my leisure activities.	*Gosto das minhas atividades de lazer* .
24	My leisure activities absorb or get my full attention.	*Minhas atividades de lazer recebem toda a minha atenção* .
25	I do leisure activities frequently.	*Pratico atividades de lazer com frequência* .
26	Given a choice I would increase the amount of time I spend in leisure activities.	*Se eu pudesse escolher, aumentaria o tempo que passo praticando atividades de lazer* .
27	I buy goods and equipment to use in my leisure activities as my income allows.	*Conforme minha renda permite, compro produtos e equipamentos para usar nas minhas atividades de lazer* .
28	I would do more new leisure activities if I could afford the time and money.	*Faria mais atividades de lazer novas se tivesse mais tempo e dinheiro* .
29	I spend considerable time and effort to be more competent in my leisure activities.	*Dedico bastante tempo e esforço para ser mais competente nas minhas atividades de lazer* .
30	Given a choice I would live in an environment or city which provides for leisure.	*Se pudesse escolher, viveria em um lugar ou uma cidade que oferece mais opções de lazer* .
31	I do some leisure activities even when they have not been planned.	*Pratico algumas atividades de lazer mesmo quando elas não foram planejadas* .
32	I would attend a seminar or a class to be able to do leisure activities better.	*Assistiria a um seminário ou uma aula para poder praticar melhor as atividades de lazer* .
33	I support the idea of increasing my free time to engage in leisure activities.	*Apoio a ideia de aumentar minha disponibilidade de tempo para participar de atividades de lazer* .
34	I engage in leisure activities even when I am busy.	*Envolvo-me em atividades de lazer mesmo quando tenho pouco tempo disponível* .
35	I would spend time in education and preparation for leisure activities.	*Dedicaria tempo em educação e preparação para as atividades de lazer* .
36	I give my leisure high priority among other activities.	*Dou prioridade para o lazer entre as minhas outras atividades* .

In the items considered equivalent, the experts also made suggestions, such as modifying the expression “make good use” to “a smart way to use” in item 1, as it helps understanding and is authentic to the original version; replacing the expression “time flies” with “time goes by fast” in item 13, avoiding the use of figures of speechin an academic instrument; remove the word “absorb” in item 24, keeping only “receive” for better understanding; replace “I do” with “ I perform” in items 25, 31 and 32; change the expression “when I am busy” to “when I have little available time” in item 34, since this interpretation of “busy” in the Brazilian context refers to the availability of time.

In the pre-test with 36 older people from UnATI, all instruments were self-applied and the average time to complete them was approximately eight minutes, with a minimum of 4 and a maximum of 20 minutes. Regarding the sociodemographic characteristics of the participants, 24 (66.7%) were women; aged between 60 and 81 years old, with an average of 71.5 years (SD=5.17); 17 (47.2%) were married or in a domestic partnership; 24 (66.7%) lived with family members; 17 (47.2%) had 12 years of education or more and 13 (36%) had 4 to 11 years of education; and 21 (58.3%) had an income of two minimum wages or more (minimum wage = R$ 954.00).

Regarding the older adults' evaluation about the instrument, all respondents considered that the directions and the answer options were easy to understand, 33 (91.7%) reported understanding of all items, 35 (97.2%) considered all items important and six older people suggested improvements for the instrument. Three participants reported difficulty in understanding items 16, 27, 30, 32, 33 and 35, but when approaching them individually, items 30, 32, 33 and 35 were correctly interpreted by these elderlies and assessed themas appropriate. Item 16 was adapted, replacing the phrase “I can be myself” with “I feel comfortable with myself”, as well as item 27, in which the sentence “as my income allows” was placed in the beginning of the sentence.

In addition, one participant requested examples of leisure in the directions, which was not possible to meet, since the definition of leisure is subjective for each individual; and one respondent asked if it was wrong to have chosen the same answer for all items, which led to the addition of the phrase “There are no right or wrong answers” in the statement.

When evaluating the 36 items, the LAM-BV showed an adequate internal consistency, with a Cronbach's alpha coefficient (α) of 0.95. The individual analysis of the components resulted in α=0.88, 0.92 and 0.88 for the cognitive, affective and behavioral domains, respectively. Table 2 shows the correlation between items and the internal consistency of each domain.

**Table 2:** Correlation between items and α by domain of LAM if the item is excluded, according to a pretest with the older population. Maringá, Paraná, Brazil, 2018 (n = 36).

Itens		Correlation between items	α
Cognitive domain			
	1	0.35	0.89
	2	0.48	0.88
	3	0.51	0.88
	4	0.67	0.87
	5	0.73	0.87
	6	0.43	0.89
	7	0.66	0.87
	8	0.58	0.88
	9	0.73	0.87
	10	0.68	0.87
	11	0.64	0.87
	12	0.63	0.88
Affective domain			
	13	0.60	0.92
	14	0.56	0.92
	15	0.68	0.91
	16	0.53	0.92
	17	0.66	0.91
	18	0.64	0.92
	19	0.72	0.91
	20	0.84	0.90
	21	0.68	0.91
	22	0.66	0.91
	23	0.73	0.91
	24	0.75	0.91
Behavioral domain			
	25	0.73	0.87
	26	0.59	0.88
	27	0.34	0.89
	28	0.68	0.87
	29	0.57	0.88
	30	0.63	0.88
	31	0.64	0.88
	32	0.69	0.87
	33	0.74	0.87
	34	0.60	0.88
	35	0.57	0.88
	36	0.73	0.87

LAM = Leisure Attitude Measurement.

Note: α: Cronbach's alpha coefficient with values above 0.7 was considered ideal.

It was observed that item 1 had the lowest (r = 0.35) correlation between items in the cognitive domain, and item 27 the lowest (r = 0.34) correlation between items in the behavioral domain. However, when excluding these items, the gain in reliability is little, as the α coefficient for each of the domains goes from 0.88 to 0.89. In the affective domain, however, no item showed a correlation between items less than 0.5. Therefore, in both situations, it was decided to keep all items of the instrument.

## DISCUSSION

This research introduces to the Brazilian context the possibility of evaluating the older population's attitude towards leisure, using a reliable instrument, even though the results deal with a preliminary version. Evidence on attitude and leisure can contribute to the analysis of the socio-cognitive processes associated with involvement in leisure, which allows the development of interventions to promote positive attitudes in this sense ^20^ .

LAM-BV showed satisfactory equivalences and adequate CVI compared to the original version. The content validation of the instrument in the aspects presented is valued as it associates qualitative strategies, by assessing equivalence and quantitative, as well as by calculating the CVI ^22^ .

The LAM-BV translation and cross-cultural adaptation process were successful after the necessary grammatical and cultural adjustments. The two Chinese versions of LAM, the first reduced, with 24 items ^16^ and the second following the same standards as the original version ^17^ , were translated separately by two bilingual Chinese scholars, who later discussed the adequacy of the terms of the two versions. These were reviewed by a third party, a Chinese scholar and, finally, the three scholars jointly developed the final version of the instrument. In the Turkish version ^21^ , LAM kept the same structure as the original version and was translated by two bilingual researchers in the field of physical education and sports and back-translated into English by a specialist.

The adaptation process is broad and does not consist of a simple translation, therefore, the expert committee must be made up of professionals with enough training, qualification and experience to make judgments and make the right decisions, considering the responsibility to create the pre-final version of the instrument ^22^^,^^25^ . The original version of LAM was evaluated by 31 specialists in the field of leisure and social psychology, and it was considered acceptable ^15^^,^^20^ .

The conception of the final version is achieved through the pre-test, when it is possible to assess the clarity of the adapted instrument, identify and review unsuitable terms that may cause confusion ^26^ . In the validation study of the LAM version for use by Malaysian university students, the authors referred to a pre-test with 105 students to obtain adequate reliability, however, they pointed out that the deeper meanings of certain items may not have been found accurately in the target language ^18^ .

Despite the current use of LAM in different contexts, most scientific publications in this area are aimed at adolescents and university students. A study carried out with Portuguese people aged 14 to 19 years showed that the attitude related to leisure is a significant predictor of satisfaction in leisure ^27^ . The results of three other recent studies carried out with undergraduate students in South Africa ^28^ , Turkey ^29^ and China ^30^ showed, respectively, that the attitude related to leisure is influenced by sociodemographic characteristics; engineering and physical education students have a more positive attitude to leisure than students from other undergraduate courses; and the attitude related to leisure reflects two new concepts, namely, prioritization and justification of leisure.

As for the older population, LAM was applied in the south of Brazil, in the version adapted to European Portuguese, with residents of long-term institutions ^13^ and older adults in the community ^6^ . In both studies, a predominantly positive attitude towards leisure was obtained, with the behavioral domain showing the lowest average ^13^^,^^6^ , results similar to the validation studies of LAM in Portugal ^19^^,^^20^ . The LAM also was applied in a survey conducted with retired Americans, showing that the attitude related to leisure is a resource that influences the way the person deals with retirement ^4^ . A Portuguese study ^10^ that involved the use of LAM revealed that older people with higher levels of education and with a more positive attitude related to leisure had better psychological well-being.

The internal consistency of the LAM-BV was adequate and the findings show the reliability of the original instrument, which has a Cronbach's alpha coefficient of 0.94 for the global instrument and 0.91, 0.93 and 0.89 for the cognitive, affective and behavioral domains, respectively ^15^ . In the other versions adapted for other countries, Cronbach's alpha coefficient ranged from 0.88 to 0.97 for the global instrument ^19^^,^^20^^,^^21^ ; from 0.81 to 0.94 for the cognitive domain; from 0.77 to 0.92 for the affective domain; and from 0.76 to 0.91 for the behavioral domain ^16^^,^^17^^,^^19^^,^^20^ .

In different applications, a lower Cronbach's alpha coefficient was observed in the behavioral subscale. The authors of the original version ^15^ already attributed this situation to the greater heterogeneity in the content of the items in this domain. Cronbach's alpha coefficient indicates the homogeneity of a psychometric instrument and is one of the most used statistical tests in cross-cultural adaptation research conducted by nursing in Brazil ^22^ . Their scores range from 0 to 1, but values above 0.7 ^14^ are considered acceptable.

Some limitations can be pointed out in this study, such as the reduced number of specialists participating in the committee, which may have overestimated the results; and carrying out the pre-test with a specific sample of older people, which may not have portrayed the difficulties in understanding by the older adults in the community.

LAM-BV may be incorporated in research and clinical practice nursing in different contexts in the health in Brazil, considering that the involvement in leisure depends on intersectoral actions. Although the psychometric analysis of the instrument is extremely important to ensure its validity, the present study represents a first step in the possibility of comparing the attitude of the Brazilian older adults related to leisure with individuals from other cultures. Furthermore, the production of standardized information in this regard in future studies will allow the development of retirement planning programs with significant involvement in leisure, aiming at a better quality of life for the older population.
